# Electronic Scooter Versus Non-electronic Scooter Injuries: A Study Comparing Distal Radius Fracture Open Reduction and Internal Fixations in an Urban Trauma Centre

**DOI:** 10.7759/cureus.93689

**Published:** 2025-10-02

**Authors:** Liang Zhi Wong, Anthony Kinnair, Chirag Rao, Ademola Adejuwon, Alistair R Hunter

**Affiliations:** 1 Trauma and Orthopaedics, University College London Hospital, London, GBR; 2 Peripheral Nerve Injury, Royal National Orthopaedic Hospital, London, GBR

**Keywords:** acute trauma care, distal radius fracture management, e-scooter injury, open reduction with internal fixation, polytrauma

## Abstract

Background

City centres in the UK have seen a gradual rise in electric scooters (E-scooters) as a form of transport and recreation. With this, accidents related to E-scooters have also risen dramatically worldwide, with an increasing number of studies exploring the profile of injury presentation and comparing E-scooters to similar modes of transport such as bicycles, but literature on this is still sparse and generalised. Despite this, there has been little increase in the regulations pertaining to the use of E-scooters.

This study compares the characteristics of E-scooter and non-E-scooter-associated injuries in patients with distal radius fractures (DRFs) treated by open reduction internal fixations (ORIFs) at our local trauma centre.

Methods

A retrospective analysis of operatively treated DRFs was conducted between January 2014 and December 2021. Electronic patient records were reviewed for patient demographics, injury characteristics and management details. Radiographs were reviewed for fracture patterns.

Results

Three hundred and ninety DRF ORIFs were performed over the study period, with a significant increase in the percentage of DRF ORIFs caused by E-scooter injuries. Five incidents occurred in 2021, representing 7% of all DRF ORIFs that year. There were no significant differences in age (mean age: 36.2 for E-scooters vs. 41 for non-E-scooters) and gender between E-scooter-related and non-E-scooter-related DRFs.

Patients with E-scooter-related DRFs treated by ORIFs were associated with a significantly greater proportion of polytrauma than those with non-E-scooters (p<0.001). A greater proportion of E-scooter riders also required a second operation (p<0.001) and longer hospital stay (p<0.001).

Conclusion

The incidence of E-scooter-related DRF ORIFs has rapidly increased at our centre in recent years. These patients’ injuries are associated with greater polytrauma rates and longer hospital stay than non-E-scooter injuries, resulting in a greater toll on patients and health services. Clinicians treating trauma should be aware of the higher likelihood of multiple injuries in patients presenting after E-scooter injuries when compared to non-E-scooter injuries and be astute in examining for them. Policymakers, on the other hand, should be aware of the financial and societal costs of these injuries to enact stricter legislation.

## Introduction

Since their introduction in 2017, electronic scooters (E-scooters) have become increasingly popular worldwide [1]. In the United Kingdom (UK), since a trial ‘green initiative’ to help mitigate limited public transport capacity was initiated in June 2020, the country has legalised the usage of E-scooters on public roads and cycle pathways for citizens over 16 years of age (Department for Transport, 2022). The original proposal suggested that increasing the usage of E-scooters allowed for transportation in a socially distanced setting during a period of national lockdown [2]. It was also intended to help reduce the burden on public transport and the number of car journeys made in urban centres [3]. 

In order to be used on public roads, E-scooters must be rented from a government-approved rental company. They must not exceed the speed limit of 15.5 miles per hour (mph), and all users must have a valid driver’s licence [4]. However, the lack of regulation regarding the use of safety equipment, poor public awareness about such laws and the rising use of privately bought E-scooters (some with top speeds of 70 mph) have led to widespread concern over their safety profile [5].

E-scooter-related injuries have increased dramatically across Europe and the United States (US), with injuries ranging from minor lacerations and contusions to major head injuries and fractures [6-9]. According to the Department for Transport UK, it was estimated that there were 931 casualties in accidents involving E-scooters from June 2020 to 2021. Seven hundred and thirty-two of these casualties were riders, and three resulted in fatalities [10]. London is responsible for 61% of all injuries despite representing just 13% of the population [11], with distal radius fractures (DRFs) representing a common fracture pattern.

There have already been several studies detailing the epidemiology of E-scooter-related injuries and evidence that their usage is associated with an increased incidence of orthopaedic-related injuries [11-14]. Some studies have also characterised E-scooter injuries and compared them to bicycle-related trauma, which is a well-studied mode of transport, and is comparable to E-scooters [6,15,16]. However, this area of literature is sparse, and existing papers tend to look at all types of injury presentations.

We aim to add to existing literature by comparing the characteristics of E-scooter and non-E-scooter (bicycle, moped and motorbike) related injuries in a Level 2 Trauma Centre, specifically looking at DRFs which are surgically managed through open reduction internal fixation (ORIF). We chose DRFs because they are either the most common or second most common fracture in cycling and E-scooter related injuries in city centres, and ORIFs as surgical management tends to have a greater socioeconomic impact on both the patient and medical services than conservative management [6,7,16,17]. This will help to better evaluate the trend of E-scooter accidents as well as their impact on health services.

## Materials and methods

A retrospective case analysis was performed on cases from January 2014 to December 2021 in our Unit, a Level Two Trauma Centre in an urban setting. Data of patients seen on the acute trauma and orthopaedic take were taken from trauma and theatre lists found on the unit’s electronic health records (EPIC, USA). The lists were then stratified to identify any patients who underwent surgical repairs of DRFs. All patients were assessed for the mechanism of injury and their involvement in vehicular accidents. Patients were then further subdivided into either E-scooter-related or non-E-scooter-related injuries. Non-E-scooter vehicles consist of bicycles, mopeds and motorbikes, which are, together with E-scooters, two-wheeled micromobility vehicles, all commonly involved in commuting-related injuries.

The data extracted was based on demographics and anthropometrics, details of the injury and then operative management. This consisted of age, gender, date of injury, mechanism of injury, pedestrian/rider status and associated injuries (including head injuries). Operative technique involved looking into the approach utilised, such as the flexor carpi radialis (volar) approach or a dorsal approach, and whether the patient required further operations. Polytrauma was described as the presence of more than one injury on presentation. 

Information regarding characteristics and severity of the injuries sustained was gathered from electronic patient notes and clinic letters. Radiographs were reviewed by the research group using Picture Archiving and Communication Software (PACS). For the patients identified, injury severity score (ISS), documentation of helmet usage and speed of travel were noted. ISS is a method of analysing the severity of injuries in polytrauma, by taking the most severe injury from each region of the body and grading from no injury to unsurvivable injury to generate a score. Fracture pattern analysis was conducted using either plain radiographs or computerised tomography with associated consultant radiologist reports. Fractures were categorised into intra- and extra-articular. Any debatable patterns were assessed by an orthopaedic consultant.

All operation notes relating to the DRF were reviewed and data were analysed, assessing the type of procedure, tourniquet usage, post-operative radiographs, and post-operative follow-up. All patients were assessed for the requirement of hospital admission, length of stay and follow-up at one year post-injury. Hospital approval was obtained for this study in accordance with hospital policies. Data were then tabulated into Excel for Mac, version 16.69 (Microsoft Corporation, USA). 

Statistical analyses were performed using IBM SPSS Statistics for Windows, Version 25 (Released 2017; IBM Corp., Armonk, New York, United States) and Excel. Descriptive statistics are presented as frequency (N), mean (SD) or number with percentage. A two-tailed P <0.05 by chi-squared (χ2) test for categorical variables or t-test for continuous variables was considered significant. Data were then re-tabulated into Excel for analysis and graphical depictions were created using the same programme. 

## Results

Three hundred and ninety DRF ORIFs were performed over the study period, of which nine involved E-scooters and 58 involved non-E-scooter vehicles. Patient and injury characteristics are shown in Table 1.

**Table 1:** Patient and Injury Characteristics of E-Scooter and Non-E-Scooter Riders Undergoing DRF ORIFs. Table discussing the patient and injury characteristics of both E-scooter and non-E-scooter riders undergoing DRF ORIFs. This includes age, whether the injured was a rider or pedestrian, mechanism of injury, gender, fracture pattern, trauma type, surgical approach, complications and follow-up. 9 of the participants who required DRF ORIFs were involved in E-scooter incidents and 58 were not. The average age in years column describes the average age and the standard deviation. DRF: Distal radial fracture; ORIFs: open reduction internal fixations

Characteristics	E-Scooter (n (%))	Non-E-Scooter (Number (%)])
Average Age in Years (SD)	36.2 (17.1)	41 (15.5)
Status		
Rider	8 (88.9)	54 (93.1)
Pedestrian	1 (11.1)	4 (6.9)
Mechanism of injury		
Fall-off	7 (77.8)	53 (91.3)
Road traffic accident	2 (22.2)	5 (8.7)
Gender		
Male	8 (88.9)	38 (65.5)
Female	1 (11.1)	20 (34.5)
Fracture pattern		
Intra-articular	7 (77.8)	45 (77.6)
Extra-articular	2 (22.2)	13 (22.4)
Trauma type		
Polytrauma	5 (55.6)	4 (6.9)
Single injury	4 (44.4)	54 (93.1)
Surgical approach		
Volar	8 (88.9)	55 (94.9)
Dorsal	1 (11.1)	3 (5.1)
Complications		
Second operation required due to polytrauma	3 (33.3)	0
No complications	4 (44.4)	53 (91.3)
Average duration of hospital stay (SD)	3.1 days (2.8)	1.2 days (0.8)
Follow-up		
Ongoing	2 (22.2)	10 (17.2)
Discharged	6 (66.7)	42 (72.4)
Lost to follow-up	1 (11.1)	6 (10.3)

**Figure 1:**
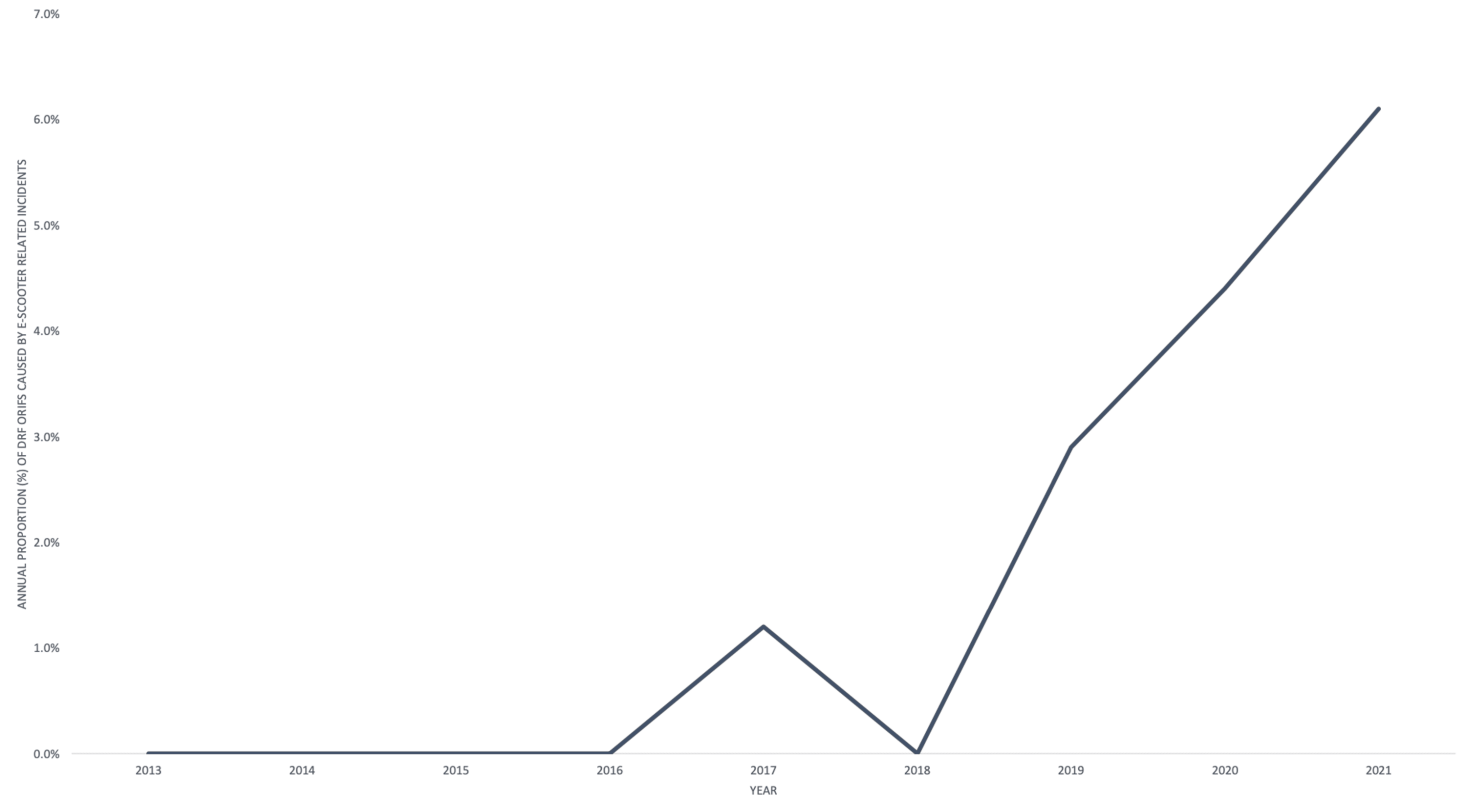
Annual Proportion (%) of Distal Radius Fracture Open Reduction and Internal Fixation Caused by E-Scooter-Related Incidents. A graph showing the annual increase in the proportion of distal radius fractures caused by E-scooter-related incidents, which required open reduction and internal fixation.

Since 2017, there has been a steady and rapid increase in the percentage of DRF ORIFs caused by E-scooter injuries. The first recorded incident was in 2017 and five incidents occurred in 2021, representing 7% of all DRF ORIFs that year (Figure 1).

There was no significant difference in the age (t (65) = -0.92, p=0.36) and gender (χ2 (1, n=67) = 0.20, p=0.65) of patients involved in E-scooter and non-E-scooter accidents. There was also no seasonal variation in E-scooter accidents, with five of the nine incidents occurring in the winter months of October to February.

**Figure 2:**
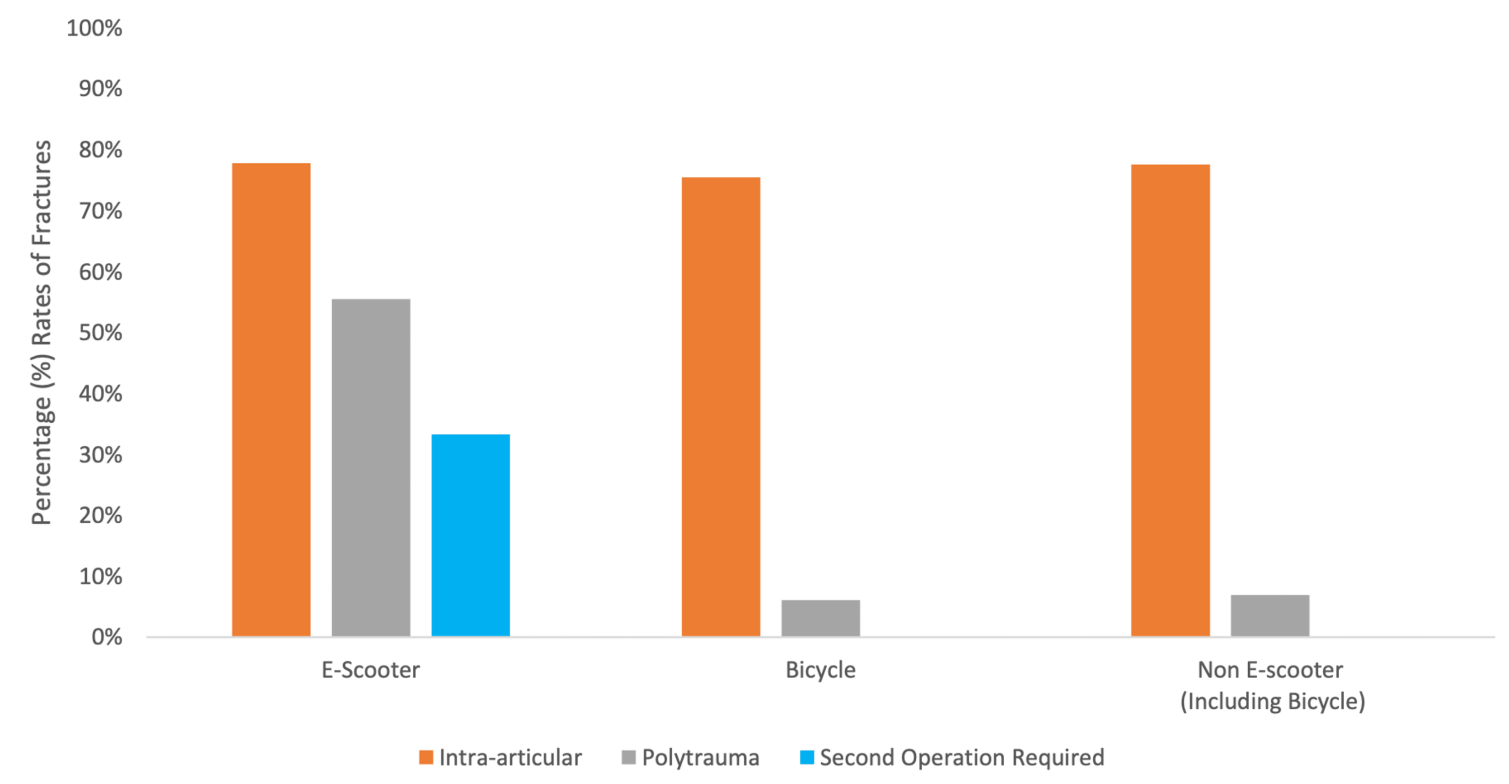
Percentage Rates of Intra-Articular Injuries and Polytrauma in E-Scooters, Bicycles and Non-E-Scooter Incidents Bar charts showing the percentage of intra-articular, polytrauma and fractures that require a second operation caused by E-scooters, bicycles and Non-E-scooter-related incidents

There were no significant differences in the proportion of intra-articular injuries in E-scooter-related DRFs compared to non-E-scooter DRFs (χ2 (1, n=67) = 0.00, p=0.99). E-scooter DRFs had a significantly greater proportion of concomitant injuries to other areas of the body, compared to non-E-scooter DRFs (χ2 (1, n=67) = 15.9, p<0.001). Concomitant injury characteristics are shown in Table 2. A significantly greater proportion of E-scooter DRFs required a separate operation due to polytrauma than non-E-scooter DRFs (χ2 (1, n=67) = 20.2, p<0.001) (Figure 2). 

**Table 2:** Concomitant Injury Characteristics of E-Scooter and Non-E-Scooter Riders Undergoing DRF ORIFs. Table showing the concomitant injury characteristics that occurred in E-Scooter and Non-E-Scooter Riders Undergoing DRF ORIFs. Five of the participants were involved in E-Scooter-related injuries and 4 were involved in Non-E-Scooter-related injuries. This includes radial head, olecranon, calcaneal, carpal bone and frontal sinus fractures. DRF: Distal radial fracture; ORIFs: open reduction internal fixations

Characteristics	E-Scooter (n)	Non-E-Scooter (n)
Radial head (contralateral)	1	1
Olecranon (contralateral) fracture	1	0
Calcaneal (ipsilateral) fracture	2	0
Carpal bone (ipsilateral) fracture	0	3
Frontal sinus fracture	1	0

The average length of hospital stay was significantly longer for E-scooter-related DRFs compared to non-E-scooter DRFs (t (65) = 4.35, p<0.001). However, there was no significant difference in the percentage of E-scooter-related DRFs which were still being followed-up one year post-operatively compared to non-E-scooters (χ2 (2, n=67) = 0.14, p=0.93).

Of all the E-scooter-related incidents, 44.5% described the rider wearing protective gear. 44.5% reported that the E-scooter was travelling over the regulated speed limit of 15.5mph at the time of the accident. This made up 80% of all polytrauma patients, all of whom required a second surgery. There is a strong positive correlation between polytrauma and travelling over the regulated speed limit at the time of the accident (Cramer’s V = 0.8).

## Discussion

There was no significant difference in the age and gender of E-scooter and non-E-scooter patients, although most E-scooter riders were aged between 20 and 29 (44%), while non-E-scooter riders were aged between 30 and 39 (24%). There was also no significant difference in gender ratios of E-scooter and non-E-scooter patients; however, both groups were predominantly male (88.9% and 65.5% respectively). This is in keeping with previous studies, which reported E-scooter riders to be generally young men aged between 20 and 40 [8,9,15,18]. Separately, we found that there was no seasonal variation in E-scooter incidence, which is contrary to previous studies that reported more accidents in the summer than winter months [7,15].

Our study shows an increasing incidence of E-scooter-related DRFs year on year. This comes as no surprise, as it has been well documented that these incidents have been increasing worldwide [6-9]. What was not previously described was that E-scooter accidents have a significantly higher incidence of polytrauma compared to non-E-scooter accidents. Polytrauma has been found to be associated with increased long-term physical disability and mental impairment, as well as reduced health-related quality of life and wrist function after DRFs [19-21]. Multiple operations are often required to reduce and stabilise the injuries sustained, resulting in a longer inpatient stay. This has been reflected in the study, where E-scooter accidents resulted in a longer hospital stay than non-E-scooter accidents. Most uncomplicated DRFs tend to be performed as a day case, which on average costs the National Health Service (NHS) around £2,700 [22]. However, when performed as a non-elective procedure requiring a longer stay, the average cost is around £4,800, even before factoring in the costs of a separate operation on another injury site [22]. The majority of these DRF fixations are performed for trauma, which usually requires a longer hospital stay. Moreover, longer hospital stays are well known to be associated with an increased risk of falls, risk of hospital-acquired infections and sleep deprivation, as well as physical and mental deconditioning. This means that E-scooter accidents tend to take a greater socioeconomic toll on the NHS as well as a greater physical and psychological impact on riders than other forms of accidents. 

There was a strong positive correlation between polytrauma and travelling over the speed limit at the time of the accident. It is well known that high-speed impacts from road traffic accidents are a major cause of polytrauma, and E-scooters appear to be no different [23]. Multiple papers have recommended that E-scooter speed limits be revised or reviewed due to the increased risk of injury and the severity of injuries sustained [5,7,15,24]. The results from our study agree with the existing evidence, suggesting that the increased incidence of polytrauma in the E-scooter cohort might be due in part to the lack of adherence to the national speed limit. However, we cannot be certain if this completely accounts for the significantly higher incidence of polytrauma in E-scooters compared to non-E-scooters, as there are no strict speed limits for cyclists, who make up most of the non-E-scooter cohort.

Our study found a similar incidence of intra-articular compared to extra-articular DRFs in both E-scooter and bicycle accidents. This is because the most common mechanism of injury in DRFs is a fall on outstretched hand (FOOSH). There was also no difference in the proportion of patients being followed up at one year post operation, which could mean that any long-term disability from polytrauma may not be reflected in follow-up rates.

The use of helmets in our patient population (44.5%) is significantly higher than in other studies (2-6%) [7,8,15,25]. This increased use of helmets could possibly be explained by the fact that our centre is in a large city, with more riders aware of the danger in commuting via E-scooters and taking the necessary precautions. Whether or not polytrauma in these patients was a direct result of the lack of other protective gear worn is unknown as only the use of helmets was documented.

Our study shows that E-scooter-related DRFs have a significantly greater impact on patients and medical systems compared to non-E-scooter DRFs due to the increased proportion of polytrauma. This is important for clinicians to note, as there tends to be a common perception amongst medical professionals that E-scooters and other modes of transport, such as bicycles and mopeds, are very similar vehicles; hence, injuries and fractures would present in a similar fashion. Crucially, being aware of the differences would help reduce the possibility of missing concomitant injuries in presenting patients.

The study also suggests that the speed of E-scooters at the time of collision is primarily responsible for causing polytrauma in surgically managed DRFs, which in turn leads to a greater burden on health services and society than non-E-scooters like bicycles. This would help to highlight the dangers of E-scooters to local policymakers so that stricter enforcement of regulations can be passed.

There are some limitations to our study. Our study is based at a single hospital, a Level Two Trauma Centre. Future studies should collect data from multiple Level One Trauma Centres based in large urban centres to better quantify the increasing incidence of E-scooter-related injuries and the reasons behind them. Secondly, due to data restrictions, our study only looked at DRFs up to 2021, limiting its generalisability to current E-Scooter trends and policies. Further studies should bridge the gap by including data from 2022 onwards. Thirdly, due to the nature of retrospective data collection, the lack of documentation meant that our study could not compare data on the rates of alcohol intoxication in patients. Although velocity and helmet use were extracted, these were not consistently documented; hence, we were unable to perform trauma stratification. Studies have found that E-scooter riders were more likely to be intoxicated than bicycle riders and were more likely not to be wearing a helmet [26,27]. Comparing these variables would have provided valuable insights into the impact that these factors have on DRFs sustained by E-scooters and non-E-scooter riders. Furthermore, future studies will include further description of the fracture pattern and mechanism of injury to improve reproducibility.

## Conclusions

In conclusion, the incidence of E-scooter-related DRF ORIFs has been found to be increasing rapidly year on year. There is a significantly greater polytrauma rate requiring a second operation and longer hospital stay in E-scooter-related accidents compared to non-E-scooter accidents. This will translate into a greater reduction in quality of life for patients and a greater socioeconomic burden for societies. It is likely that the number of E-scooter DRFs will continue to rise as their usage increases every year. Clinicians treating trauma should be aware of the differences in E-scooter and non-E-scooter accidents and anticipate that there will be a higher likelihood of multiple injuries in patients presenting after E-scooter injuries.
